# Editorial: Victimization in sexual and reproductive health: violence, coercion, discrimination, and stigma

**DOI:** 10.3389/fpsyg.2023.1253136

**Published:** 2023-08-08

**Authors:** Beatriz Pérez, María Teresa Ramiro, Jaime Barrientos

**Affiliations:** ^1^Departamento de Psicología, Universidad de Oviedo, Oviedo, Spain; ^2^Departamento de Psicología, Universidad de La Frontera, Temuco, Chile; ^3^Departamento de Psicología Evolutiva y de la Educación, Universidad de Granada, Granada, Spain; ^4^Facultad de Psicología, Universidad Alberto Hurtado, Santiago, Chile

**Keywords:** sexual health, reproductive health, violence, coercion, discrimination, stigma

Sexual and reproductive health is a state of physical, mental, emotional, and social wellbeing and not merely the absence of sexual or reproductive disease. This definition implies the right to have a fulfilling and secure sex life. Also, the freedom to make decisions about the functions and processes related to the reproductive system, without risks, coercion, discrimination, and violence. However, conservative and traditional social norms that mark the expected and accepted behavior, limit full development in sexual and reproductive health to individual, cultural, and structural levels. This monograph aims to address sexual and reproductive health limitations due to traditional and conservative value trends. In addition, to address the consequences of these limitations for those who, due to their condition, choice, or personal situation, challenge the normative standards of their community.

Six of the eleven articles in this monograph address reproductive health issues. Corvino and D'Andrea, in a qualitative case study of health care workers in Italy, explore the care experiences of migrant women in childbirth who may come from conservative cultural backgrounds. The results show that members of their community can be coercive in their treatment. In addition, health care protocols that are not socioculturally relevant, generated in them an experience of health care as extremely medicalized, uncomfortable, and even abusive and violent. Xie et al. conducted a systematic review of the impact of the stigma of infertility on mental health and the quality of life of infertile women. They concluded that this stigma is common among women who suffer from infertility. Negative social interaction from the partner, family or community increases the susceptibility to anxiety and depression, suffering from feelings of inferiority, loneliness and guilt.

The remaining four articles on reproductive health deal with issues related to the practice of abortion. Montero et al. and Casas et al. have conducted qualitative studies with key informants in health afield and health system managers in Chile. Montero et al. explore the exercise of conscientious objection to abortion in public institutions. They identify pernicious practices such as declaring conscientious objection without justification or the provision of dissuasive and erroneous information. They conclude that conscientious objection is a barrier to applying abortion law and a form of structural violence. Casas et al. focus their efforts on the difficulties in accessing abortion services due to rape, when the woman does not fit the precepts of the “ideal victim”. The authors conclude that in Chile's primary public health system, the breach of these rules involves denying rape survivors victim status. They are treated unfairly and revictimized, making access to abortion difficult and even impossible.

The works by Pérez et al. and Knapton et al. address topics related to community attitudes to abortion from a quantitative perspective. The study by Pérez et al. seeks to design the Community Attitude to Abortion Scale (CAAS) with a Chilean population, which has obtained adequate psychometric properties. The participants who identify with socially conservative religious or political groups show more stigmatizing attitudes to abortion and less agreement with women's empowerment. They also questioned other rights or freedoms, such as euthanasia, LGBTIQ+ rights, or feminism. For their part, Knapton et al. address the radicalization against abortion in the US. They conclude that those participants in the minority regarding the state's opinion on abortion experience greater social exclusion and support for extreme actions. This relation is mediated by need-threat and group identity.

Four of the remaining five articles deal with topics related to sexuality and traditional gender norms from a quantitative perspective. Gómez-Berrocal et al. analyze the association between different profiles of adherence to the sexual double standard (SDS) and individual and contextual variables in the Spanish population. They consider the SDS as a gender bias on sexual behaviors. They conclude the need for a multilevel approach to study this phenomenon, emphasizing aspects such as the framework of relationships between men and women, endogroup favoritism, and the cultural and normative context. Likewise, Orellana et al. identify four profiles of heteronormativity according to the degree of essentialism and normative behavior in Chilean University students. Among the findings, it stands out that, although lesbian, gay, bisexual, and queer participants fit a low level of heteronormativity profile, they also form a profile that adheres to heteronormative behavior without endorsing essentialist beliefs. The authors explain that this may be due to contextual factors and cognitive-cultural schemata.

On the other hand, using mediation models, Sepúlveda-Páez et al. and Tao et al. have studied topics related to the perceived sexual stigmatization in men who have sex with men and its effect on behavioral variables. Sepúlveda-Páez et al. examine internalized homophobia as a risk factor for developing risky sexual behaviors in a Chilean sample. The results include an indirect and inverse effect of internalized homophobia mediated by sexual self-efficacy regarding “having multiple sexual partners”. In China, Tao et al. explore the mediating role of self-efficacy and social support in the association between HIV stigma and HIV self-management behaviors such as daily physical health practices. There are no direct influences, but they find significant indirect effects in single and chain mediation models. Both works highlight the importance of considering mediating variables in the examined effects and their importance in understanding the phenomena.

Finally, Stockman et al. performed a systematic meta-review on the impact of sexual violence from an ecological perspective. The results show that sexual violence is associated with multiple types of damage and negative consequences, although also with positive changes. The authors conclude that the aftermath of sexual violence involve a complex interaction of risk and protective factors at multiple levels. These must be considered for a complete understanding of the phenomenon and approach to intervention with survivors. However, they denounce the lack of studies integrating macro-level factors, such as rape myth acceptance to subcultural level.

This Research Topic, therefore, generates new contributions regarding the weight of traditional and conservative cultural norms on the full development of sexual and reproductive health. This is because the authors who contribute to this monograph: report on the potential pernicious role of health professionals in the service and application of the Law when such norms guide behavior; provide a new scale to measure community attitudes to abortion and evidence of the radicalization process in minority opinion groups against abortion; address the consequences of stigma perceived on health and self-care behaviors, like the stigma of infertility, internalized homophobia, and HIV stigma, underscoring the importance of mediating variables for a greater understanding, such as self-efficacy or social support; and they develop typologies based on the assimilation of cultural norms regarding gender and the expression of sexuality, like the SDS and heteronormativity. Definitely, this Research Topic advances understanding of the issue since the studies presented make a substantial contribution to making it visible, understanding the effects and scope of traditional cultural societies on sexual and reproductive health, and producing evidence to mitigate those effects.

## Author contributions

All authors listed have made a substantial, direct, and intellectual contribution to the work and approved it for publication.

